# Variation in predator species abundance can cause variable selection pressure on warning signaling prey

**DOI:** 10.1002/ece3.315

**Published:** 2012-07-16

**Authors:** Janne K Valkonen, Ossi Nokelainen, Martti Niskanen, Janne Kilpimaa, Mats Björklund, Johanna Mappes

**Affiliations:** 1 Centre of Excellence in Evolutionary Research, Department of Biological and Environmental Science, University of JyväskyläJyväskylä, Finland; 2 Animal Ecology/Department of Ecology and Genetics, Evolutionary Biology Centre (EBC), Uppsala UniversityUppsala, Sweden

**Keywords:** Aposematism, predation, selection, snake, viper, warning signal

## Abstract

Predation pressure is expected to drive visual warning signals to evolve toward conspicuousness. However, coloration of defended species varies tremendously and can at certain instances be considered as more camouflaged rather than conspicuous. Recent theoretical studies suggest that the variation in signal conspicuousness can be caused by variation (within or between species) in predators' willingness to attack defended prey or by the broadness of the predators' signal generalization. If some of the predator species are capable of coping with the secondary defenses of their prey, selection can favor reduced prey signal conspicuousness via reduced detectability or recognition. In this study, we combine data collected during three large-scale field experiments to assess whether variation in avian predator species (red kite, black kite, common buzzard, short-toed eagle, and booted eagle) affects the predation pressure on warningly and non-warningly colored artificial snakes. Predation pressure varied among locations and interestingly, if common buzzards were abundant, there were disadvantages to snakes possessing warning signaling. Our results indicate that predator community can have important consequences on the evolution of warning signals. Predators that ignore the warning signal and defense can be the key for the maintenance of variation in warning signal architecture and maintenance of inconspicuous signaling.

## Introduction

Various conspicuously colored animals advertise their defense to potential predators with bright colors. For example, many toxic poison frog and butterfly species exhibit bright warning coloration (Poulton 1890; Cott 1940). The conspicuousness of warning signals enhances predator avoidance as improved detection and recognition facilitate predator learning. Warning signals can thereby be expected to evolve toward conspicuousness (reviewed in Ruxton et al. 2004). However, not all defended prey species advertise themselves to predators by having overtly conspicuous coloration (Endler and Mappes 2004). In their model, Endler and Mappes (2004) showed that “weak” warning signals can evolve and be maintained if predators vary in their willingness to attack defended prey. In other words, if some predators are able to cope with the secondary defenses of conspicuous prey, predation pressure should increase due to detectability of the prey and lead to selection for reduced conspicuousness. An example of this is suggested in the seemingly inconspicuous pine sawflies (*Neodiprion sertifer* and *Diprion pini*) that are preyed upon by both ants and great tits (*Parus major*), although they are chemically defended and seemingly not palatable food for birds (Lindstedt et al. 2011).

Indeed, most prey species are preyed upon by more than one predator species and predators may vary in their willingness to attack defended prey. Thus, in theory, fitness of warning signals may depend on the given predator community structure (Endler and Mappes 2004; Mappes et al. 2005; Nooan and Comeault 2009; Mochida 2011), however, studies quantifying this in natural predator communities are lacking.

**Figure 1:**
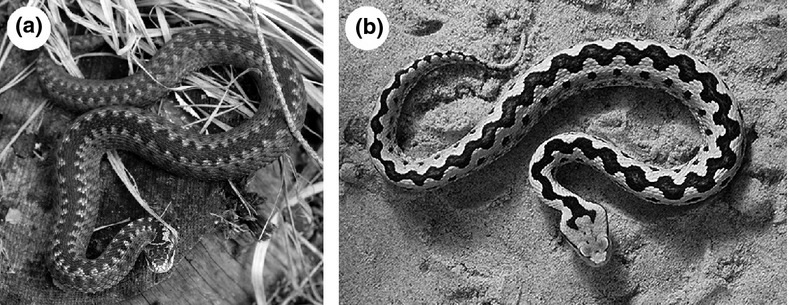
European vipers (*Vipera* sp.) exhibit characteristic dorsal zigzag pattern which is shown to act as a warning signal for avian predators. However, despite the signaling function of the zigzag pattern some species like *Vipera berus* (a) are seemingly inconspicuous whereas others like *Vipera latastei* (b) exhibit more conspicuous coloration.

Several species of European vipers (genus *Vipera*) seem to exhibit rather inconspicuous coloration despite being venomous (De Smedt 2001; Fig. 1a). They share a characteristic dorsal zigzag pattern (see De Smedt 2001), which has been suggested to offer protection through camouflage by hindering detection by predators (Andrén and Nilson 1981). However, more recent studies have shown that the zigzag pattern of vipers acts as a warning signal despite its seemingly inconspicuous nature. Studies by Wüster et al. (2004), Niskanen and Mappes (2005), and Valkonen et al. (2011a,b) have demonstrated that zigzag patterned snakes are preyed upon less by avian predators than snakes without a zigzag pattern, indicating that the zigzag pattern is in fact aposematic. However, these studies do not take into account regional variation in predation pressure or its relationship with local predator community structure. Niskanen and Mappes (2005) found large variation in predator pressure among locations which may indicate that local predator community structure can have a significant effect on strength and direction of predation. Here, we assess if variation in composition of predatory community structure affects the predation on warningly and non-warningly colored snakes. We combined data collected during three field experiments by Valkonen et al. (2011a,b) to test whether the abundance of natural predator species is related to the benefits of warning signaling.

## Materials and methods

We observed birds of prey during three different experiments conducted in Coto Doñana National Park, Southern Spain during springs 2008–2010 (Valkonen et al. 2011a,b). Experiments spanned approximately 25% of the 6794 hectare reserve. Following previously employed methods, (Andrén and Nilson 1981; Brodie 1993; Pfennig et al. 2001; Wüster et al. 2004; Niskanen and Mappes 2005) artificial snake replicas with zigzag (wide and narrow) and other patterns (plain, striped, or blotched) were placed in the field in transect lines and imprints on the replicas caused by avian predator attacks were observed. Two to five different pattern types of snakes were used in each transect lines and length of the transect lines varied from 300 to 750 m (20–50 snake replicas). Snake replicas were placed in 15 m intervals and the number of differently colored replicas were balanced within transect. Artificial snakes were made of gray pre-colored plasticine (CaranD'Ache, Modela Noir) and had patterns painted on them with black paint. As we did not find any effect of the background matching of the snake replicas in the previous experiment (Valkonen et al. 2011a; but see also Wüster et al. 2004; Niskanen and Mappes 2005) background effect is not included in data presented here. For more detailed descriptions of the methods and coloration of snake replicas, see Valkonen et al. (2011a,b). Only attacks caused by birds were included in the analyses because mammals might recognize artificial prey items from olfactory cues and be attracted by the odor of plasticine (Rangen et al. 2000; Valkonen et al. 2011a,b; Valkonen and Mappes 2012). During our experiments, we often observed mammalian predators (e.g., red fox, *Vulpes vulpes*) following our tracks along the transect lines and non-selectively biting almost all snake replicas in the area. Furthermore, we were not able to estimate the number of preying mammals in the study areas.

During the experiments, raptors flying over the experimental areas were surveyed using a telescope and binoculars. The duration of observations varied from 55 to 75 min and the observations were repeated one to three times at each transect line in different days between 10 am and 3 pm. Thereby our data does not consider possible owl attacks. However, the only relevant owl in the area is the barn owl (*Tyto alba*), which is almost exclusively a rodent predator with only few observations on other prey of which few, except small lizards, are reptiles (Herrera 1974). The most commonly sighted raptor species were black kite (*Milvus migrans*), red kite (*Milvus milvus*), booted eagle (*Hieraaetus pennatus*), common buzzard (*Buteo buteo*), and short-toed eagle (*Circaetus gallicus*) (Table 1). These five species are likely to be the most important avian predators of snakes in the area and herein we consider them as key predators. These species are different in their foraging behavior and food choice. The short-toed eagle is highly specialized in preying upon snakes and its diet consists mainly of snakes and lizards (Forsman 2007). The diet of the common buzzard consists mainly of small mammals and birds, but they are also known to commonly consume snakes (Selas 2001; Forsman 2007). The diet of the booted eagle consist mainly of medium-sized birds, big lizards, small mammals (Forsman 2007), and snakes (Valkonen et al. 2011a). Black and red kites are more generalist, feeding on carrion, small mammals, birds, insects, reptiles, and fish (Forsman 2007).

**Table 1:** Number of observed raptors of each species/observation time (h)

Species	Max observations/h	Mean observations/h	SE
Black kite *(Milvus migrans)*	68	22.20	3.33
Red kite *(Milvus milvus)*	27	5.16	1.06
Booted eagle *(Hieraaetus pennatus)*	14	4.13	0.54
Common buzzard *(Buteo buteo)*	7	0.95	0.24
Short-toed eagle *(Circaetus gallicus)*	2	0.60	0.11
Common kestrel *(Falco tinnunculus)*	2	0.16	0.07
Western marsh-harrier *(Circus aeruginosus)*	1	0.06	0.03
Imperial eagle *(Aquila adalberti)*	1	0.06	0.04
Peregrine falcon *(Falco peregrines)*	1	0.03	0.02
Lesser kestrel *(Falco naumanni)*	1	0.01	0.01

In all three experiments, bird observations were conducted at a total of 40 transect lines and 1443 snake replicas. Based on findings of previous experiments (Wüster et al. 2004; Niskanen and Mappes 2005; Valkonen et al. 2011a), snake replicas were divided into two categories: (1) Aposematically colored snake replicas, which included all zigzag patterned replicas (*n* = 722); and (2) non-aposematic, which included plain, striped, and blotched patterns (*n* = 721). The abundance of each raptor species in each location was calculated by dividing the total number of observed individuals of each species by total observation time. A generalized linear mixed model (GLMM) with binomial distribution was used to analyze the data. As a response variable we used fate of each snake replica (attacked or not). As time that transect lines were in the field varied from 41.92 to 73.67 h (mean = 52.25 h), we corrected our response variable by the catching effort (binomial trial with 1 or 0 attack out of hours that transect line was in the field). Coloration of the snake replica and the abundance of each key raptor species were used as explanatory variables. To account for the sampling structure of our data (six of 40 transects were conducted in the same location in consecutive years) we included “year” and “location” as random effects in our model. The model selection procedure started from the model including all possible two-way interactions of raptor species and snake replica coloration then simplified. Model selection was based on significance of the terms in the model (Table 2). Statistical analyses were conducted using R 2.11.1 and lme4 package.

**Table 2:** Generalized mixed model selection. Response variable is the fate of the individual snake replica balanced by times that transect line was in field (catching effort)

Model	df	AIC	χ^2^	sig. χ^2^	*Z*	sig. *Z*
1 ∼A^*^B+A^*^ML+A^*^MI+A^*^H+A^*^C+1|Y+1|L	14	645.88				
2∼MI+A^*^B+ A^*^ML+A^*^H+A^*^C+1|Y+1|L	13	643.95	0.078	0.78	−0.271	0.78
3∼MI+ML+A^*^B+A^*^H+A^*^C+1|Y+1|L	12	642.12	0.166	0.68	0.405	0.69
4∼MI+ML+C+A^*^B+A^*^H+1|Y+1|L	11	641.41	1.293	0.26	−1.118	0.26
5∼MI+ML+C+H+A^*^B+1|Y+1|L	10	641.33	1.925	0.17	1.398	0.16
6 ∼MI+ML+C+A^*^B+1|Y+1|L	9	639.34	0.003	0.96	0.057	0.95
7∼MI+C+A^*^B+1|Y+1|L	8	637.35	0.009	0.92	−0.151	0.88
8∼C+A^*^B+1|Y+1|L	7	636.95	1.606	0.21	−1.519	0.13
9∼A^*^B+1|Y+1|L	6	637.60	2.651	0.10	1.64	0.10

Abbreviations of the explanatory variables are: A, coloration of snake replica (aposematic or not); B, abundance of common buzzard; ML, abundance of red kite; MI, black kite; H, booted eagle; C, short-toed eagle; Y, year; L, location. Asterisk indicates interaction term of the variables and + indicates main effects. If interaction term is indicated also main effect is included. χ^2^ value and significance level of χ^2^ indicates change from higher model. *Z* value and its significance are for significance of the removed term in the higher model. Model selection was based on significance of the terms in the model.

## Results

**Figure 2:**
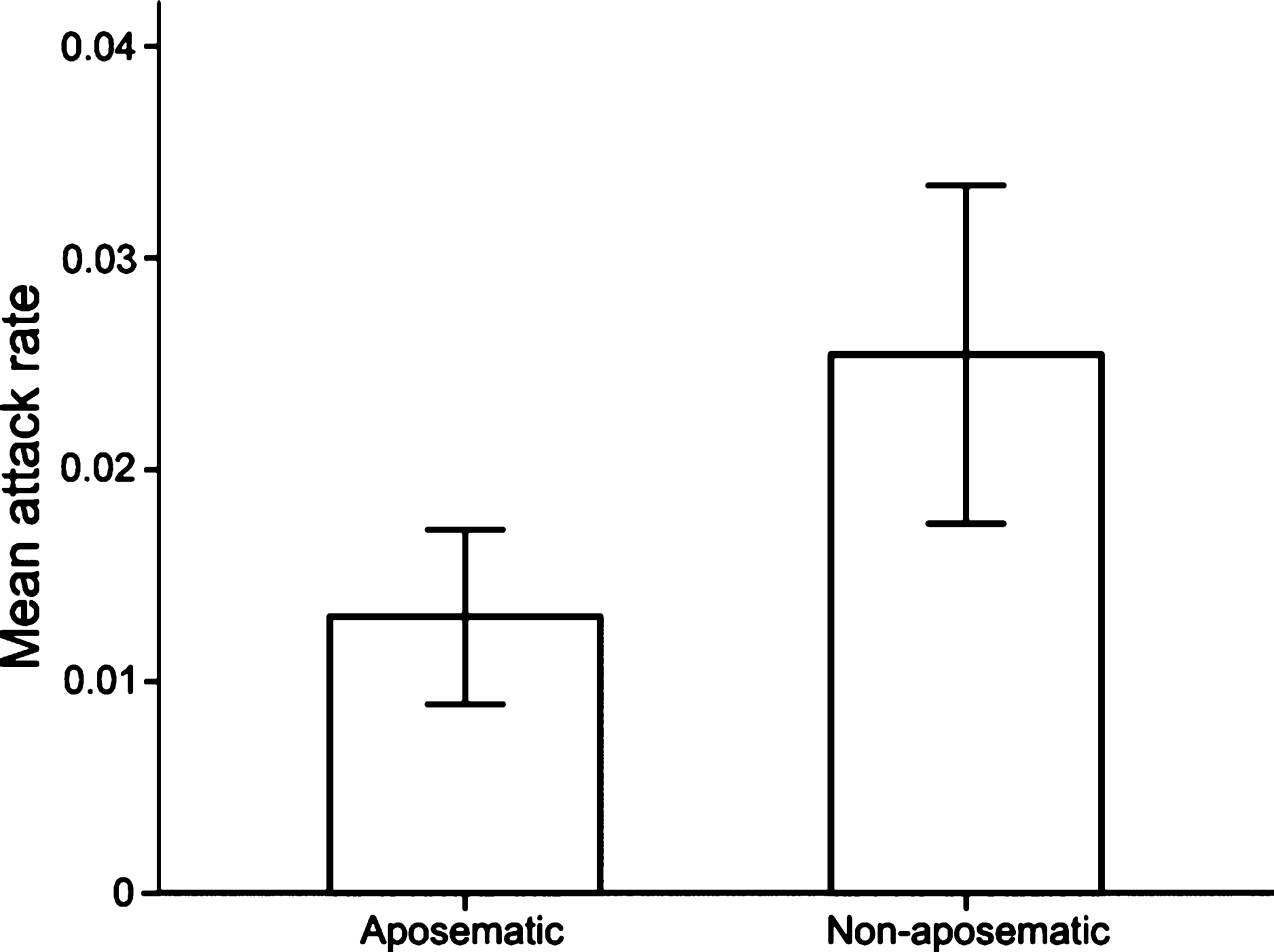
Avian attack rates (attacks in 10 h/snake replica) on aposematic (zigzag) and non-aposematic (plain, stripe, or disruptive pattern) snakes. Bars represent 95% confidence interval.

**Figure 3:**
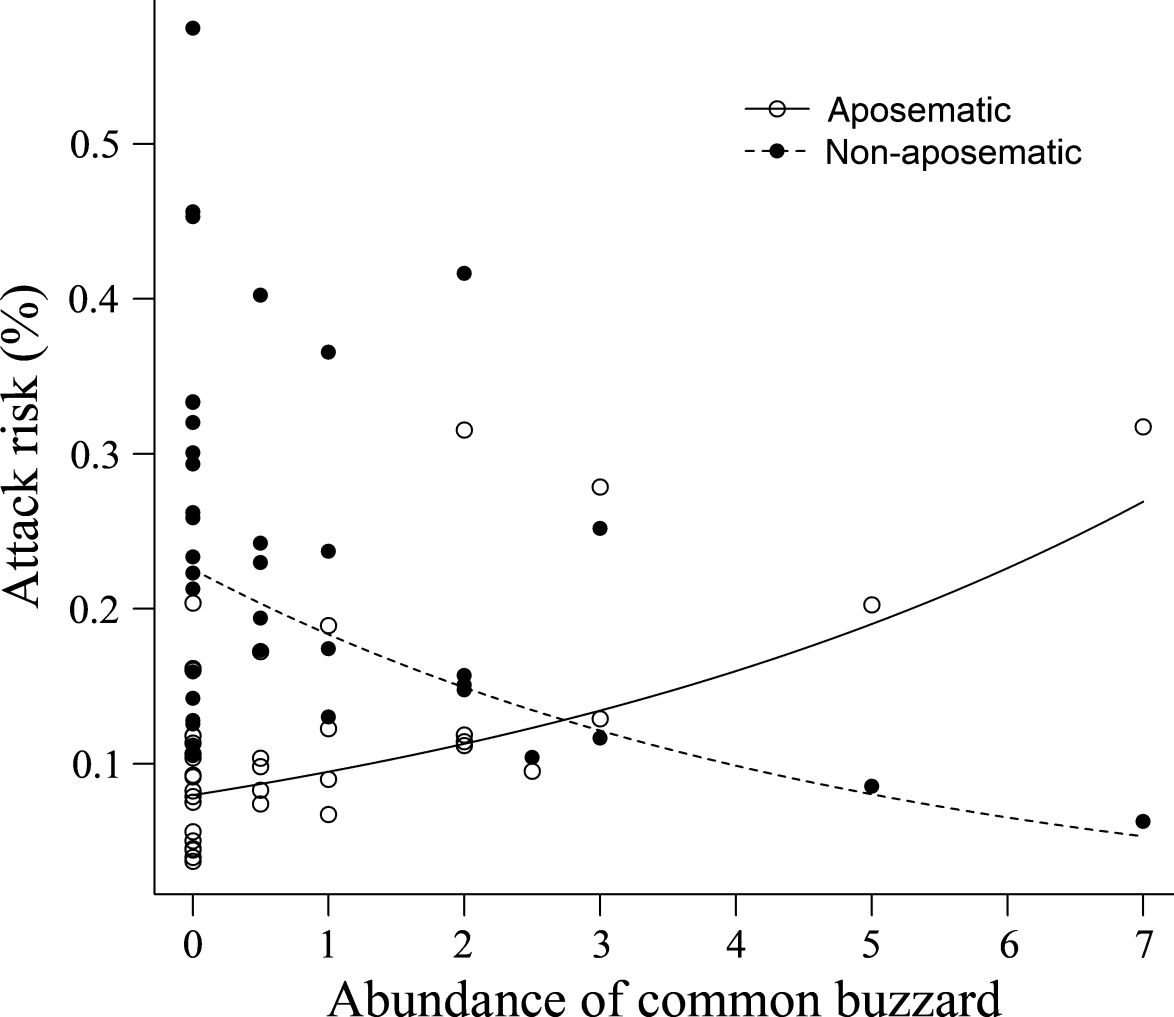
Attack risk of aposematic (open dots) and non-aposematic (closed dots) snakes related to abundance of common buzzard. Lines represent model estimates (solid line, aposematic; dashed, non-aposematic snakes).

The overall predation pressure on aposematically colored snake replicas was lower than the predation pressure on non-aposematic snake replicas (*Z* = −4.56, *P* < 0.001) (Fig. 2). The only raptor species that caused significant deviation from the general trend of lowered predation on aposematic snakes was the common buzzard (Table 3). The predation on snake replicas increased by function of the interaction of aposematic coloration of snake replicas and abundance of common buzzard (Z = 2.47, *P* = 0.013). In other words, if common buzzards were abundant there was a higher probability of attack on aposematic snakes possessing warning signals compared with non-aposematic snakes (Fig. 3). We did not find significant interactions between snake replica coloration and abundance of black kites, red kites, or booted eagles, which indicates that these species generally do avoid warningly signaling vipers (Table 2).

**Table 3:** Generalized linear mixed model (model 9 in Table 2) fitted in predation data. Dependent variable, number of attacks on snake replicas is balanced by time. Year and location is included in the model as random effects

Terms in the model	Estimate	SE	*Z*	*P*
Intercept	−6.11	0.23	−25.72	<0.001
Aposematic	−1.04	0.22	− 4.56	<0.001
Common buzzard	−0.21	0.13	−1.54	0.125
Aposematic × Common buzzard	0.38	0.15	2.47	0.013

## Discussion

Overall, aposematically colored snakes suffered less avian predation than non-aposematic snakes. However, supporting the hypothesis that predator species do vary in their tendency to attack warningly colored prey, we found significant interaction of prey coloration and abundance of only the common buzzard. More importantly, if common buzzards were abundant enough, the probability to get attacked by raptor was higher among aposematic snakes compared with non-aposematic ones. This suggests that the abundant occurrence of specialized predators may cause local selection pressure to favor a less conspicuous warning signal within a prey population. Although predator community structure has been suggested to affect the benefits of conspicuous warning signaling of a prey population (Endler and Mappes 2004; Nooan and Comeault 2009; Mochida 2011), these suggestions are so far based on theoretical models or observations on predation pressures in different locations without detailed data about predatory community structure. Empirical results presented here with more detailed natural predator community data provide rare support that abundant occurrence of specialist predators may select reduced conspicuousness of warning signal.

Avian predators that are specialized snake predators are expected to not hesitate to attack vipers. Although the common buzzard is not considered to be a snake specialist, this species is known to commonly attack snakes, including vipers (Selas 2001; Forsman 2007). Common buzzard is also the only raptor species that significantly decreased survival advantage of warningly colored snake replicas in our data. Surprisingly, the abundance of short-toed eagles did not significantly affect the survival advantage of warningly colored snake replicas because this species is known to be a highly specialized snake predator (Forsman 2007). It is possible, however, that the effect of the short-toed eagle cannot be observed in our data due to their low abundance (Table 1). Alternatively, short-toed eagles are shown to prefer larger prey than we used in these experiments (Gil and Pleguezuelos 2001) and they might have ignored our snake replicas. Abundance of more generalist predators; black kite, red kite, and booted eagle did not cause significant deviation on the general trend of the attack probabilities. The fact that these species did not have significant interaction with prey coloration indicates that they generally do avoid preying on warningly colored snakes. By avoiding attacking conspicuously signaling vipers, generalist raptors may favor protective coloration of the local viper species (*Vipera latastei gaditana*). As being very abundant in Southern Spain, these generalists may cause seemingly conspicuous warning coloration of *V. l. gaditana* compared with most European vipers (see De Smedt 2001; Fig. 1b).

Besides the fact that several predators can learn to recognize and avoid aposematic species, many predators can also learn to kill and handle defended prey (Skelhorn and Rowe 2006). If a predator is capable of handling defended prey without extra costs, the direction of selection toward conspicuous warning signal can disappear or reverse. In addition, there is experimental evidence that several characteristics, not only the conspicuous colors of prey animals can be recognized and avoided by predators. For example, natural predators are shown to avoid triangular head shape of vipers (Valkonen et al. 2011b), dragonflies (*Aeshna grandis*) can recognize and avoid body shape of wasps (Kauppinen and Mappes 2003); body shape and size of the prey has been observed to affect foraging behavior of praying mantis (*Sphodromantis lineola*) (Prete 1990). Such features can thereby serve a signaling function to predators without increased cost of conspicuousness.

Prey animals are subjected to predation by several predators which can wary their behavior and cognitive capabilities. Data presented here provide further support for the idea that some players of the natural predatory community can cause selection pressure that leads to reduced conspicuousness of aposematic signal. According to our results, conspicuous warning signals can be expected to evolve in the locations where majority of predators avoid local warning signal. Whereas in locations where increasing abundance of predators that do not hesitate attacking on signaling prey, costs of conspicuousness may lead the evolution of moderate or less conspicuous warning signaling.
